# Exploring user perspectives on SMART: qualitative study of novel digital intervention targeting metabolic care in schizophrenia and related disorders

**DOI:** 10.1192/bjo.2025.10954

**Published:** 2026-01-20

**Authors:** Urska Arnautovska, Gabrielle Ritchie, Rebecca Soole, Andrea Baker, Nicole Korman, Agata Bialkowski, Dan Siskind, Alyssa Milton

**Affiliations:** Faculty of Health, Medicine and Behavioural Sciences, The University of Queensland, Brisbane, Australia; Metro South Addiction and Mental Health Services, Brisbane, Australia; Queensland Centre for Mental Health Research, Brisbane, Australia; Business School, The University of Queensland, Brisbane, Australia; Faculty of Medicine and Health, The University of Sydney, Sydney, Australia; ARC Centre of Excellence for Children and Families over the Life Course, Brisbane, Australia

**Keywords:** eHealth, mHealth, psychosis, mobile phone, metabolic syndrome

## Abstract

**Background:**

Effective implementation of novel digital technologies to improve health outcomes requires an in-depth understanding of end-users’ perspectives and experiences.

**Aims:**

We sought the perspectives of people with schizophrenia and schizophrenia-related disorders (SSD) on the acceptability of a novel short text message-delivered intervention targeting metabolic health, called Schizophrenia and diabetes Mobile-Assisted Remote Trainer (SMART).

**Method:**

Twenty-nine participants with SSD and either at risk of type 2 diabetes (T2D) or with T2D, were recruited from 3 mental health sites in Brisbane, Australia. They were provided, for 12 weeks, with SMART text messages that embedded psychoeducation and behaviour change techniques to facilitate lifestyle behaviours crucial for metabolic health. Interviews explored participants’ experiences of SMART, barriers to its use and suggestions for improvement. The qualitative data were collected by three mental health researchers and analysed using thematic analysis.

**Results:**

Three themes were generated: acceptability and user experience, feasibility and implementation considerations, and mechanisms supporting change. SMART was found to be highly accessible and engaging, and participants reported positive lifestyle changes, including healthier eating, increased physical activity, weight loss and smoking cessation. The messages reinforced learning and supported participants’ readiness for change.

**Conclusions:**

SMART is a world-first digital intervention aimed at improving metabolic health and diabetes self-management in individuals with SSD. High levels of acceptability of SMART highlight its strong potential as a digital innovation that can support its users in protecting their metabolic health while limiting the detrimental side effects of antipsychotic medications.

People living with schizophrenia and schizophrenia-related disorders (SSD) experience a higher prevalence of metabolic risk factors including obesity,^1^ suboptimal diet and sedentary lifestyle,^2^ as well as lack of health education,^3^ contributing to high rates of type 2 diabetes (T2D)^4^ and cardiometabolic-driven mortality.^5^ High risk of T2D is further compounded by the metabolic side-effects of antipsychotic medications that lead to rapid weight gain, dyslipidaemia and insulin resistance.^5^ Despite the well-known cardiometabolic burden, metabolic health-focused approaches tailored for people with SSD are lacking. To address existing limitations of in-person lifestyle approaches – such as low engagement and high dropout rates^6^ – the Lancet Commission on improving physical health in people with severe mental illness^5^ recommends delivering diabetes prevention lifestyle programmes using digital tools. This reflects a growing body of evidence supporting the feasibility^7,8^ and potential efficacy of digital interventions for improving mental health-related outcomes in people with SSD.^7^ However, while emerging mobile health (mHealth) interventions – those delivered via mobile technologies such as smartphone apps, wearables and text messages – have demonstrated feasibility in improving individuals’ health outcomes,^9^ there is a dearth of qualitative research that could help in understanding end-users’ experiences of novel metabolic health-focused interventions. This represents a critical gap, because the recently updated Lancet Commission on implementing lifestyle interventions within mental health care^10^ emphasises the importance of involving end-users during both the intervention development and testing stages, to optimise the uptake and acceptability of new approaches. This study, therefore, explored the experiences of people with SSD in using a novel, co-designed digital intervention targeting metabolic health, the Schizophrenia and diabetes Mobile-Assisted Remote Trainer (SMART).^11^

## Method

### Design

This study was part of a larger, mixed-methods study that explored the acceptability, feasibility and preliminary efficacy of SMART over 12 weeks. The current study presents results based on interviews with participants who used SMART, in addition to their usual treatment, for 12 weeks.

### Participants and recruitment

Participants were recruited from three sites within the Metro South Addiction and Mental Health Services, in metropolitan Brisbane, Australia, including an out-patient endocrinology clinic embedded within a community mental health clinic and two residential rehabilitation mental health facilities (community care units), between September 2024 and January 2025. Following referrals from the treating clinician who identified potential participants based on the study inclusion/exclusion criteria, the research staff contacted the individual to organise a face-to-face meeting where potential participants were provided with detailed information regarding the study, and conducted the screening/eligibility assessment and consent process. Participants were included if they were: at least 18 years old; had a diagnosis of SSD as per DSM-5; at risk of T2D (i.e. had a diagnosis of metabolic syndrome (MetS) or pre-diabetes), or had T2D; able to use a mobile phone; able to read/understand English; and able to provide informed consent.

Verbal consent was witnessed and formally recorded in an individual’s participant information and consent form by obtaining their signature. The authors assert that all procedures contributing to this work comply with the ethical standards of the relevant national and institutional committees on human experimentation, and with the Helsinki Declaration of 1975 as revised in 2013. All procedures involving human subjects were approved by the Metro South Human Research Ethics Committee (no. HREC/2023/QMS/100469).

### Intervention

The intervention SMART was co-designed with experts from a range of disciplines, including psychiatry, psychology, endocrinology, nutrition and mHealth, as well as people with lived experience of schizophrenia/schizophrenia-related conditions and T2D, through an iterative, multi-phase process (published previously^11^) to improve metabolic health in people with SSD who are at risk of developing T2D, and to improve self-management of T2D in those with existing T2D. SMART provides users with personalised and semi-interactive psychoeducation and behavioural nudges delivered via a purpose-built message distribution system. The content of SMART text messages is informed by the Social Cognitive Theory^12^ and Self-Determination Theory (SDT),^13^ while each text message incorporates relevant behaviour change techniques (BCT) selected from the BCT taxonomy (V1).^14^ The core modules (topics) – offered to all users – target nutrition, weight management, physical activity and stress coping, while optional modules, which users can choose if relevant to them, target smoking/vaping cessation and blood glucose level monitoring. Personalised elements include text messages addressing an individual by their preferred name, choosing their preferred time of the day to receive messages, ranking the core modules in order of importance for their health and choosing one/both/none of the optional modules. Having obtained consent to participate, the research staff set up an individual’s profile in the secure online system, initiating a 12-week distribution of personalised text messages. The frequency of the text messages depended on an individual’s ranking, with a total of six per week (the schedule and frequency of messages are provided in Supplementary Table 2 available at https://doi.org/10.1192/bjo.2025.10954). An additional text message was sent weekly for either of the two additional modules, if an individual requested this. Possible replies were ‘Yes’, ‘No’ and ‘Unsure’, with a response prompting an automatic reply (distinct for ‘Yes’ and ‘No/Unsure’), which may have included a hyperlink to a website relevant to the original message content (e.g. recipes for a healthy diet).

### Data collection

Data pertaining to demographic and clinical characteristics of participants were collected at baseline, while exploration of participants’ experiences of the intervention was assessed through semi-structured interviews conducted at the 12-week end-point, which were audio-recorded with the participant’s permission. The interviews were conducted face-to-face, at a participant’s residence, by U.A., G.R. or R.S., who are all practising psychologists and experienced in conducting interviews for research purposes with people living with a severe mental health condition. The interview questions asked about the perceived usefulness of the SMART intervention, barriers to its use and suggestions for improvement, and were guided by the key implementation outcomes of the acceptability, appropriateness and feasibility of the intervention. The full interview guide is provided in Supplementary Material 1. Participants were reimbursed for their time in completing assessments (AUD $50 gift voucher for baseline and AUD $50 gift voucher for end-point).

### Data analysis

Qualitative responses were transcribed verbatim by R.S., with a minority by G.R., and subjected to thematic analysis using an inductive approach^15^ by R.S. in collaboration with G.R. and U.A., through an iterative process. Data management and coding were conducted in NVivo (version 14 for Windows, Lumivero, QSR International, Denver, Colorado, USA; https://www.qsrinternational.com/nvivo-qualitative-data-analysis-software/home), following these steps: transcription of audio-recordings, familiarisation with the data through iterative reading, initial identification of codes, organisation into broader thematic categories and refinement of final themes. The research team engaged in regular discussions to ensure consistency in coding and theme development, to enhance the credibility and relevance of findings. Scores on the three questions evaluating the implementation of the intervention, assessed within the interview, were aggregated and are presented as mean scores.

### Reflexivity of practice

The research team consisted of eight members: U.A., G.R. and R.S. (psychologists, collected and interpreted the data); A. Baker (nurse, clinical trial manager); N.K. and D.S. (psychiatrists); A.M. (psychologist, informed the interview guide); and A. Bialkowski (lived experience of schizophrenia). All contributed to study conceptualisation, reflexivity discussions and manuscript preparation. U.A., G.R. and R.S., all practising clinicians and experienced in qualitative research and reflexive methods, worked actively to minimise bias and ensure that participants’ voices were accurately represented. Aware of their clinical psychology backgrounds and involvement in co-designing the SMART intervention, they kept reflexive journals and held regular debriefings with senior clinicians (N.K. or D.S.) for external perspectives. A. Baker, who managed the study and conducted clinical assessments over 12 weeks, offered additional insights based on her ongoing interactions with participants. To further reduce researcher bias and incorporate lived experience, five randomly selected transcripts were reviewed by A. Bialkowski and discussed with U.A., to ensure that themes and interpretations accurately reflected participants’ experiences. These lived-experience insights are detailed in Supplementary Material 2.

## Results

Twenty-nine participants were eligible and consented to participation; 41% were female with a median age of 44 years. All had a diagnosis of SSD, and just over half had a diagnosis of T2D. Participant characteristics at baseline are presented in Table 1. Two participants voluntarily dropped out during the study period: one in week 4, due to familiarity with the provided information, and one in week 11, due to worsening of their psychosis symptoms. As such, interviews were conducted with the remaining 27 participants, producing in total 426.6 min of interview data. Each interview ranged in length from approximately 7.0 to 27.3 min, and data saturation^16^ was determined within the eighth transcript.

**Table 1:** Participant characteristics at baseline (*N* = 29)

Characteristics	*n* (%)
Gender	
Male	17 (58.6)
Female	12 (41.4)
Living situation	
Supported housing	8 (27.6)
Independent (living alone)	12 (41.4)
Independent (living with others)	9 (31.0)
Education	
Year 10 or below	7 (24.1)
Years 11–12	10 (34.5)
Higher education degree	12 (41.4)
Metabolic health status	
T2D	17 (58.6)
Pre-diabetes	2 (6.9)
Metabolic syndrome	17 (58.6)
Smoking status	
Never	11 (37.9)
Former	5 (17.2)
Current	13 (44.8)
Cognition	
Normal cognition	14 (48.3)
Mild cognitive impairment	9 (31.0)
Moderate cognitive impairment	6 (20.7)
	Median (IQR)
Age, years	44 (36–52)
Weight, kg	104.6 (96.3–124.9)
Body mass index^a^	37 (30.7–41.8)
Cognition	
Total MoCA score	25 (21–27)

T2D, type 2 diabetes mellitus; IQR, interquartile range; MoCA, Montreal Cognitive Assessment.

a. Body mass index ≥30 is classified as obesity.^17^

**Fig. 1:**
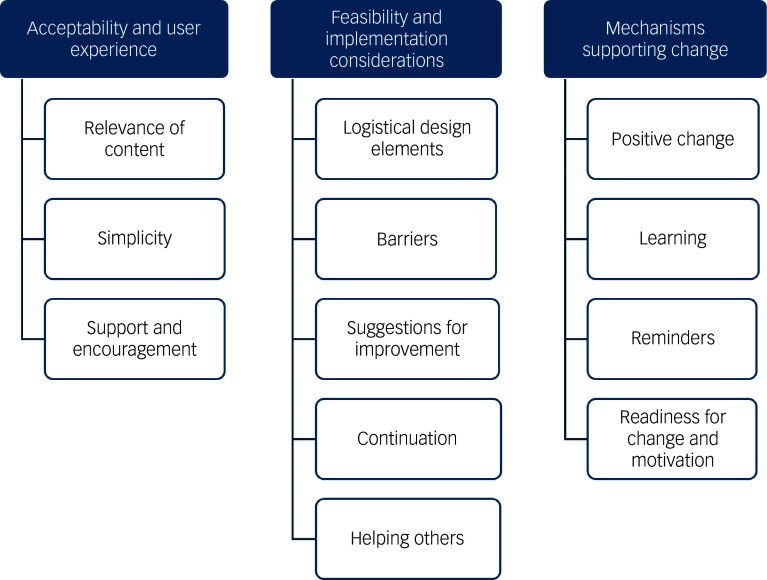
Three key themes and the sub-themes emerging from thematic analysis of the interview data.

Three overarching themes were identified (Fig. 1): (a) acceptability and user experience, (b) feasibility and implementation considerations and (c) mechanisms supporting change. Specific subthemes were derived inductively from participants’ responses, capturing the nuances of their experiences and perspectives, with direct quotes supporting the themes (Supplementary Table 1). Additionally, suggestions for improvement of the SMART intervention were identified from across the three themes and are presented in Table 2.

**Table 2:** Recommended changes to the delivery and design of the SMART intervention based on participants’ experiences

Recommendations and example quote
Personalise the number of text messages
‘4–6 per week were too many; it can feel like homework’ (Participant 29, female, age 29, T2D) ‘Needs another message to help with tie in… Sometimes you’ve forgotten about the lunchtime… Maybe two texts of like 8:30 a.m. and maybe around lunchtime to remind me’ (Participant 5, male, age 43, T2D) ‘Nothing comes to mind rather than like maybe a second topic of the day. Like a second link to a different topic. Or asking if it wants to continue same topic’ (Participant 24, male, age 27, MetS)
Complement text messages with paper-based information
‘I would have liked if all the information had been in one. In a book, it would have been easier to go to, you know, if the text said open up page 63, it would be easy to open that up and read it on paper… That would have made a lot more enjoyable. Then I’d use it more’ (Participant 2, female, age 49, pre-T2D)
Include more recipes, possibly within text messages
‘More recipes to give you the idea. Maybe practical cooking lessons with it… That there would be a link to how to do it’ (Participant 28, female, age 52, MetS)
Offer an optional video to demonstrate physical activity exercises
‘I’m asking other people and learning from other people who have travelled a similar journey… And you can see [via] the YouTube video’ (Participant 12, male, age 38, T2D and MetS)
Provide an option to make it more interactive
‘If you could even after that reply, still reply something and kept on going the communication… it’d be good if I could ask questions. And then, like, they’d respond with, like, more information’ (Participant 7, male, age 23, MetS)
Offer text messages for a longer period and follow-up
‘I’m thinking if it went longer… Maybe six months… ‘cause, I reckon it’s a good time for change… You could then implement those changes’ (Participant 10, female, age 57, T2D) ‘Or could you get permanently sent the messages and the government should do [that]’ (Participant 9, male, age 46, pre-T2D) ‘A follow up… In two months time… [to check] if you’re cooking properly, if you exercise [could be over the phone]’ (Participant 29, female, age 29, T2D)

T2D, type 2 diabetes; MetS, metabolic syndrome.

### Theme 1: acceptability and user experience

Participants described their perceptions of engaging with SMART in terms of content relevance, simplicity and its role as a source of encouragement and support. The content of the messages was generally perceived as highly relevant to both participants’ overall well-being and, more specifically, to their metabolic health, as expressed by one participant:

‘I think it was relevant to the dealing with diabetes and trying to lose weight. So, it did help go sort of a spark the thought process’. (Participant 5, male, age 43, T2D).

Several participants also perceived the diversity of topics as positive and engaging; for example:

‘I liked how it was something different each time, so one day would be like weight loss and then another day it would be like diet’. (Participant 7, male, age 23, MetS).

The ability to rank the different modules in order of priority for an individual’s health appeared to strengthen the utility of the intervention, by allowing participants to focus on topics that were most important to them. The majority of participants also acknowledged the simplicity of SMART’s design, highlighting that the language in the messages was easily understandable and ‘more engaging just because it’s so simple’. The usability of SMART was also related to the simplicity of engaging (responding to the text messages), as one participant said:

‘It takes 10 seconds, just yes or no answer. That’s how quick and easy it is’. (Participant 17, male, age 44, T2D)

Nearly all participants noted that SMART text messages were beneficial to their well-being, describing them as a source of ‘support’ (including social support through phone check-ins by the researchers), ‘validation’ and a sense of ‘going in the right direction’, as well as fostering a feeling that ‘someone cared’. This sense of connection facilitated a common experience of SMART text messages being ‘encouraging’, contributing to participants’ active engagement (for one participant, responding to messages was described as ‘a privilege’) and their overall enjoyment of the intervention. The aspect of support was substantially important to the participants and was expressed as discussing with their support workers, family members or friends about topics in text messages; receiving general support from them when mentioning the SMART text messages; prompting the participants to initiate collaborative meal planning or engagement in exercise; and providing a stable supportive system during difficult times, such as admission to hospital due to worsening of psychosis symptoms.

While all participants reported having had a very positive experience of using SMART, a few suggested improvements to encourage its use and optimise its benefits. These recommendations are summarised in Table 2.

### Theme 2: feasibility and implementation considerations

Participants discussed the practical aspects involved in delivering and using SMART, which included both the enablers and potential barriers to implementation. Nearly all participants endorsed the feasibility and appropriateness of the message length, their frequency and delivery format, finding them well suited to their needs (‘I wouldn’t, honestly ask to change it’). For example, one participant said:

‘For me, it was the fact that this wasn’t overwhelming. I think that with diabetes, there’s so much information that you got to get around your head and when it was just one message a day, that kind of just seemed achievable to be honest’. (Participant 12, male, age 38, T2D and MetS)

Another suggested that the frequency of text messages could remain constant, rather than reducing over time:

‘I liked it at the start ‘cause I think I was just excited for the messages and then I noticed that there were like slowly getting less and I was like it’s a rare commodity now’. (Participant 24, male, age 27, MetS)

The majority of participants also appreciated the ability to personalise the timing of message delivery, along with the consistency and anticipation of the message at a certain time. However, a few participants identified limited phone credit as being a barrier to responding and accessing web links embedded within the response messages, and two suggested complementing text messages with paper-based materials; for example:

‘I would have liked if all the information had been in one, in a book, it would have been easier to go to, you know, if the text said open up page 63, it would be easy to open that up and read it on paper… then I’d use it more’. (Participant 2, female, age 49, pre-T2D)

A few other participants suggested increased interactivity and customisation, such as a ‘follow-up text’, to remind them of the suggested activity or another message asking whether they would like to expand on the first topic or receive more information on a different topic.

Overall, SMART was largely seen as accessible and well structured, with the majority of participants expressing a strong willingness to recommend it to others. The predominant view was to improve their lifestyle and overall health, but also to counteract the metabolic side-effects of antipsychotic medication, ideally before developing T2D. As one participant said:

‘Because you know, the medication does weight gain and there’s only so much the doctors can do […] but having a programme like this, it helps because, not only can you look after your selfcare, but you get a few tips here and there about food and what to do. It might be good for people who are also like not social […] they feel alone and they don’t know what to do with their health or they might not have enough family members to encourage them or they might not even have friends to like, help them’. (Participant 29, female, age 29, T2D)

### Theme 3: mechanisms supporting change

Participants made direct connections between using SMART and the changes they had observed in their knowledge, motivation and health behaviours after being prompted about them in the text messages. Many participants described SMART’s lifestyle-focused content as providing them with increased awareness, new insights and practical strategies; for example:

*‘*It made me think consciously about things around to do with my diabetes. It made me feel like, what’s the word? Yeah. Just aware. And with that awareness comes, I’m able to make good decisions which help me with my diabetes management’. (Participant 12, male, age 38, T2D and MetS)

Furthermore, messages that provided tips on healthy nutrition, exercise and stress or T2D management served as ‘a good reminder’ ‘to keep on track with their health goals’ and ‘to make better decisions’. Messages were viewed by participants as empowering them with the knowledge and confidence needed to transition from contemplating a change to actually actioning the desired behaviour, supporting their readiness for change. Reflecting this increased motivation and preparedness for change, one participant referred to SMART as an ‘action-based programme’.

The majority of participants also reported making positive lifestyle changes, including choosing healthier eating options (e.g. ‘trying to incorporate more fruit and veggies’, ‘I’ve tried to stop eating cookies’); increasing their physical activity (e.g. ‘I’ve increased my exercise by double’); losing weight (e.g. ‘consistency with the size and try not to eat too much’, ‘I’ve lost 8 kg’); and one quitting smoking, attributed it to receiving SMART text messages:

‘[Quitting smoking was] something I was wanting to do for a while and then I just needed the push and like more prep. And then when I see the links that it was sending me, I was like, oh, this is really, really helpful information’. (Participant 24, male, age 27, MetS)

These positive changes further instilled a sense of accomplishment and confidence (one participant reported ‘a lot less anxiety’ while making the lifestyle changes during the 12-week period) and, for a few, also improved their general well-being; for example:

*‘*Feel a bit like happier, I think. Because I think it’s like, it’s like a process that is happening and, how do I say it like, like, it feels like I’m actually getting something done… You know accomplishing’. (Participant 7, male, age 23, MetS)

## Discussion

This study provides critical insights into users’ perspectives of a novel, world-first digital intervention targeting metabolic health that is tailored to people living with SSD. This qualitative exploration taps into the implementation aspects that are paramount for bridging digital health disparities and for further tailoring of novel digital tools like SMART for historically marginalised populations.^18^

Interviews with participants demonstrated that receiving and responding to SMART text messages, which provided psychoeducation and behavioural prompts to increase lifestyle behaviours relevant to T2D, was acceptable and contributed to perceived improvements in their mental and physical health, most likely through an increased sense of awareness, confidence and knowledge about healthy lifestyle choices.

Building on from our co-design work in developing SMART,^11^ the current qualitative exploration of SMART continues to adopt the participatory approach, embodied in models such as a generative co-design framework for healthcare innovation^19^ and the Medical Research Council framework for complex interventions, which has been used successfully in previous studies involving self-management of chronic mental health conditions.^20^ Key aspects of SMART that were perceived as being critical for participants’ continuous engagement included relevance of the content, simplicity of the digital delivery design (both in terms of length of the text messages and responding to them), along with the provision of support and encouragement that motivated the participants to move further along the behaviour change continuum, from contemplating a change to engaging in the desired behaviour.^21^ Similarly, receiving regular behavioural reminders and psychoeducation regarding diabetes prevention and self-management seem to have increased knowledge and awareness about how to protect and improve metabolic health, which further had a flow-on effect on participants’ motivation to put this new knowledge into action, generating feelings of enjoyment and accomplishment. These hypothesised mechanisms of change are in line with the premises of the SDT,^13^ where autonomous motivation (e.g. enjoyment, being aware and seeing the personal importance of the behaviour) has been shown to influence engagement in health behaviour, such as physical activity in people living with severe mental illness.^22,23^ Moreover, such motivational design principles also corroborate empirical evidence supporting the value of self-management for people with severe mental illness, who often experience a complexity of mental and physical conditions.^24^ The importance of techniques that increase motivation in people with schizophrenia has been highlighted previously^25^ and these are also incorporated as fundamental elements within other existing apps for this population group.^26^

Furthermore, in contrast to other studies using text messaging, typically with the aim of improving medication adherence,^27^ SMART allowed personalisation of the time of message delivery, as well as the frequency of text messages, through ranking four content topics in the order of their importance, which reinforced the content relevance for users. The ability to choose specific aspects of the intervention supports the autonomy and agency of individuals, a key factor in effective self-management and active involvement with care.

Participants in the current study – all clinically stable and residing within a metropolitan area in a Western context – expressed strong support for the feasibility of SMART as an adjunctive treatment approach, and reported minimal barriers. Only 2 out of 29 participants reported difficulties with replying to text messages due to lack of phone credit, which they were able to overcome within a week by purchasing more credit. No other technical difficulties were reported during the study duration. Combined with participants’ positive appraisals of the intervention simplicity, the lack of barriers to using SMART may have contributed to the majority of participants wanting to continue receiving the messages, as well as to their feeling confident that SMART would help other people with similar conditions in reducing their metabolic risk and providing protection from developing T2D. One person specifically mentioned their potential to slow the weight gain associated with antipsychotic medication side-effects, which validates the personal struggles of people experiencing unwanted antipsychotic-induced weight gain reported in a previous qualitative study.^28^ Cumulatively, these findings provide strong evidence of SMART’s feasibility, one of the key implementation outcomes within RE-AIM and other implementation frameworks.^29^

While the key objective of SMART was to improve metabolic health in people with schizophrenia, it is noteworthy that participants also perceived text messages as being supportive, non-pressuring and caring, which appeared to foster a sense of connection. It is possible that participants may also have felt cared for through taking part in face-to-face assessments at baseline and the 12-week end-point, as well as by receiving a check-in phone call at weeks 4 and 8, typically by the same person who conducted the baseline assessments. While the role of human support, integrated within digital interventions targeting people with schizophrenia, has been established previously,^30^ these findings reinforce the role of human interaction, even if this is delivered remotely over the phone. To disentangle the potential effects of conducting face-to-face assessment with the researchers, an evaluation of SMART without routine clinical care, where research interactions would be minimised, would be warranted.

Reporting of minimal suggestions for the improvement of SMART echoes experiences of other designers who advocated for a more simplistic, albeit inclusive, user-centred design of novel digital tools for people with psychosis.^31^ The improvements in the current study were expressed by only a few participants and included: a preference for a longer period of using SMART (e.g. until desired health behaviours are established); consistent (rather than reducing) frequency of text messages; an option for greater interactivity (ability to ask follow-up questions), and for a possible additional text message within the same day; as well as a paper-based resource that could complement the information in the text messages. While increasing interactivity, possibly through integration of artificial intelligence or artificial intelligence-driven chatboxes, which are becoming increasingly prevalent within digital mental health,^18^ may serve a minority of users, there is a risk of expanding the complexity of the intervention at the expense of losing the current overall acceptability and enjoyment of using SMART text messages, due to increased cognitive and technical demands.

Related to the safety of digital mental health interventions, which remains an underrepresented area of research,^32^ it is also noteworthy that none of the participants in the current study expressed concerns around privacy which, in other digital interventions delivered in this population, is a common issue. This may be due to active engagement of participants with the clinical services from which they had been recruited, including some of them with the research nurses from their participation in other, pharmacological trials. To thoroughly examine critical issues including trust, privacy or any concerns or preferences around culturally sensitive messaging, safety assessment and risk-mitigation strategies should remain integral to study design in future testing, following existing recommendations on safety in digital mental health interventions.^32^ These issues may be particularly relevant for sub-populations that may experience socioeconomic deprivation (e.g. those in the Global South); food insecurity – which is common in people with severe mental illness^33^ – in regional/remote areas where privacy is harder to maintain due to smaller population numbers; and those from culturally diverse backgrounds. Capturing a greater diversity of SMART users, in terms of their socioeconomic characteristics, would therefore be beneficial in future studies of SMART.

Similarly, further upgrades should be carefully considered and discussed with a range of potential users. In the current study, half of the participants had mild to moderate cognitive impairment (as measured with the Montreal Cognitive Assessment) and included predominantly those with treatment-resistant schizophrenia. Validation of these results among other subgroups, including those with first-episode psychosis, as well as in outer metropolitan settings such as regional/remote areas, is warranted. To address barriers to the implementation of digital mental health innovations within health services, such evaluation should follow the logic model of implementation frameworks^34^ and, specifically, include aspects of implementation research that would address how best not only to implement and deliver, but also to sustain lifestyle-focused digital intervention within routine clinical practices in a given local context.^10^ A potential adaptation towards integrating peer support should also be considered, given the positive experiences of peer-supported, self-management mental health interventions in previous studies.^20,24,35^ Importantly, tailoring implementation strategies to the unique clinical and community context in which SMART would be delivered would increase equity, as well as the likelihood of favourable outcomes for both individuals and health services.^36^

While this study was not designed to evaluate the efficacy of SMART, the majority of participants did report making changes in their diet, physical activity orweight management, with one ceasing smoking. They reported increased awareness (e.g. thinking consciously about diabetes self-management), knowledge (e.g. eating mindfully and smaller portions) and confidence in looking for additional information (e.g. by pointing them towards relevant websites), as well as a feeling of accomplishment as they started making the changes. These concepts align with the notions of self-management, recommended to manage complex mental health conditions and schizophrenia specifically,^24^ and patient activation, which relates to a person’s knowledge, skills and confidence in managing their health and healthcare and is an established predictor of lifestyle behaviours critical to metabolic health (e.g. physical activity);^37^ metabolic indicators (e.g. cholesterol and progression from pre-diabetes to T2D);^38^ T2D self-management;^39^ and health service use^40^ in the general population^41^ and those with SSD.^42^ Given that individuals with schizophrenia struggle to take an active role in managing their health, increasing one’s skills, knowledge and confidence relevant to metabolic health through SMART could have significant clinical and resource implications. The implications of this study are further reinforced by the fact that the characteristics of participants are representative of the local clinical population in terms of prevalence of the metabolic syndrome,^43^ and of the broader population of people with SSD in terms of cognitive impairment.^44^ However, it is noteworthy that the prevalence of those with T2D in this study (58.9%) is higher than that estimated in the wider population of people with SSD (18.9%).^4^

There are a few limitations of this study to consider. Several of the participants were out-patients who were known to the research team from their participation in previous clinical trials, which may have increased compliance with the intervention and favourable reflections of its usage. Replication of these findings, with minimal involvement of the researchers, in other real-word settings would, therefore, be warranted. Second, while mobile phone ownership among people with schizophrenia is increasing,^36^ the digital delivery format nevertheless excluded those who either did not own a mobile phone or were uncomfortable with using text messaging. Finally, we did not assess participants’ attitudes and beliefs about digital interventions, nor their general information technology skills, all of which may have influenced their perceptions of SMART. We also acknowledge that the duration of the interviews was generally short and, therefore, precluded a comprehensive and deep qualitative analysis of the contextual factors that may have impacted participants’ experiences; however, this was expected given the negative and cognitive symptoms often experienced by people with SSD. Importantly, an individual’s socioeconomic conditions should also be taken into consideration, particularly their access to physical activity resources and healthy food options, when examining the usefulness of metabolic health-focused interventions like SMART in real-word settings.

In sum, the findings of this qualitative analysis of 29 users of SMART living with SSD in a metropolitan area of a Westernised country are encouraging and support progression towards higher-level evidence generation through hybrid effectiveness–implementation trials with embedded safety monitoring and risk management strategies, and a focus on local service-based adaptation to maximise its impact in diverse service settings. SMART text messages were perceived as acceptable, feasible and potentially helpful in initiating changes in lifestyle behaviours critical for the prevention and self-management of T2D. Suggestions for improvement, based on the experiences of the current sample of participants, included continuation with the interventions beyond 3 months, ability to increase the frequency of text messages and greater interactivity. Potential adaptation of the intervention, however, should be carefully balanced against the risks of reduced engagement and privacy concerns, and conducted in close collaboration with end-users. Further testing of clinical efficacy, as well as of the implementation of SMART, from the perspective of both clinicians and patients and within various healthcare settings, is warranted.

## Supporting information

Arnautovska et al. supplementary material 1 Arnautovska et al. supplementary material

Arnautovska et al. supplementary material 2 Arnautovska et al. supplementary material

Arnautovska et al. supplementary material 3 Arnautovska et al. supplementary material

Arnautovska et al. supplementary material 4 Arnautovska et al. supplementary material

## Data Availability

Original interview data obtained during the current study will not be made available, due to privacy reasons.

## Supplementary material

The supplementary material is available online at https://doi.org/10.1192/bjo.2025.10954
