# Supplementary material for: Exploring user perspectives on SMART: qualitative study of novel digital intervention targeting metabolic care in schizophrenia and related disorders
Source: BJPsych Open. 2026 Jan 20;12(1):e44. doi: 10.1192/bjo.2025.10954 (PMC12835714; doi:10.1192/bjo.2025.10954)
Supplement: Arnautovska et al. supplementary material 1 — Arnautovska et al. supplementary material [file S205647242510954Xsup001.docx]

**Supplementary Table 1.** Summary of themes and sub-themes with illustrative quotes

| **Theme** | **Key reflections** | **Illustrative quotes** |
| --- | --- | --- |
| ***Acceptability and user experience*** | | |
| *This theme focuses on how participants perceived and engaged with the intervention in terms of enjoyment, ease of use, and whether participants found the content meaningful.* | | |
| Relevance of content | - Participants found the text message content engaging and relevant to both overall well-being and diabetes management. - The diversity of topics covered, along with the ability to prioritise areas of interest, enhanced the intervention’s usefulness by allowing individuals to focus on what was most applicable to their needs. | *I think it was relevant to the dealing with diabetes and trying to lose weight. So, it did help go sort of a spark the thought process (Participant 5, Male, 43, T2D)*  *Relevant to where I'm at and it progressed as day in day by day goes. Yeah, the progress. Like it flowed (Participant 21, Female, 37, T2D and MetS)*  *I liked that it was consistent. I liked how it was something different each time, so one day would be like weight loss and then another day it would be like diet, and it gives me like options on what I could do (Participant 7, Male, 23, MetS)*  *A good range. Yeah, it like covered how you're feeling during the day. Your diabetes. Where your head spaces at and all that kind of jazz so it was the right gap. Getting it on a daily basis. Just to. Just making me feel like that I'm OK (Participant 21, Female, 37, T2D and MetS)*  *Because I find like it's quite hard to find information about diabetes and I found, like those links, just took you to what sort of foods you should be eating, what sort of snacks you can be eating. Just simple things like that. That's what I found it helpful (Participant 27, Male, 51, T2D and MetS)*  *All the topics were really helpful (Participant 19, Female, 47, MetS)* |
| Simplicity | - Participants found the intervention easy to use and the language in the text messages easy to understand, describing it as straightforward and free from unnecessary complexity. - The simplicity of the design contributed to a positive user experience, making engagement with the content accessible and participation manageable. | *I think it's more engaging just because it's so simple.*  *It's easy to understand what the message is telling you (Participant 7, Male, 23, MetS)*  *It doesn't have all the bells and whistles that frankly don’t really mean much. It's very core, a very strong grounding and like I said, like a real health concern… it doesn't need to be flash, it just needs to work (Participant 11, Male, 43, T2D and MetS)*  *Like I said, it's like short messages. It's not like, you know, long paragraphs or, you know. So it’s easy to answer the questions, so it's not complicated. Simple language, yeah (Participant 14, Female, 48, T2D)*  *Well with the text message, it takes 10 seconds just yes or no answer. That's how quick and easy it is, yeah (Participant 17, Male, 44, T2D)*  *I found like it was very easy to use. I thought it was going to be difficult but it's just so, so simple (Participant 27, Male, 51, T2D and MetS)*  *Yeah, everything was seamless. Like I loved it. You know, it was easy to understand, you know, all the information was clear and accurate. You know, there's no confusion (Participant 29, Female, 29, T2D)* |
| Support and encouragement | - Participants described the intervention as a source of support and encouragement, appreciating its positive and non-pressuring approach. - Participation appeared to foster a sense of connection and care making participation feel like a privilege rather than a task which contributed to participants' overall engagement and enjoyment of the intervention. | *It was informative, OK, and it wasn't pushy with anything…. and it was positive. I like that. The way they voiced it. Yeah, but there was no oh you should do this yeah, but it was kind of like oh what do you think of this kind of approach (Participant 10, Female, 57, T2D)*  *Receiving the text messages on the morning time gives me a little bit of validation. A bit of support, awareness and just knowing that I'm in the right direction, going in the right direction (Participant 21, Female, 37, T2D and MetS)*  *I'm happy with, the text came through, it was a bit different, I was expecting more texts than I got, but I wouldn’t, honestly ask to change it… it was a privilege, answering the little text. There's nothing I didn't like about them (Participant 6, Male, 59, T2D)*  *No I'm really grateful to participate in this study. I think it it'll help a lot of people. Yeah (Participant 14, Female, 48, T2D)*  *I really liked it, I found it encouraging… it just felt like, you know, someone cared, you know (Participant 19, Female, 47, MetS)*  Oh if you say no yeah. Oh it's very encouraging. They didn't like, you know like say you know that's wrong, or you can't do that…… it was encouraging. It was like, oh. If you can't do it, that's OK. That's the message I got *(Participant 29, Female, 29, T2D)* |
| ***Feasibility and implementation considerations*** | | |
| *This theme includes practical aspects of delivering the intervention, identifying both facilitators and barriers to its use.* | | |
| Logistical design elements | - Overall, participants found the length of text messages appropriate and not burdensome. - The frequency of text messages was deemed appropriate by participants with several expressing a preference for greater frequency or the ability to stipulate a preference to maintain the same frequency of text messages [rather than receiving a reduced number of messages over the course of the intervention]. - The ability to customise the timing of message delivery to accommodate individual schedules and preferences was noted and the response format ("yes," "no," "unsure") was acceptable. - The check-in phone call was appreciated by participants as an additional reminder and source of support, facilitating modification of design elements (e.g., delivery timing, topic prioritisation) and adding further personalisation to the intervention. | *The size of the text is good. You know that much information is readable. You know when you get too much information, you don't have time to read it. Yeah, keeping it small and simple is a good way to do it. I think. I like that (Participant 2, Female, 49, pre-T2D)*  *For me, it was the fact that this wasn’t overwhelming. I think that with diabetes, there's so much information that you got to get around your head and when it was just one message a day, that kind of just seemed achievable to be honest (Participant 12, Male, 38, T2D and MetS)*  *Well, it's good 'cause then I can really think about it. I think really more than that, I would have been a bit overwhelmed (Participant 12, Male, 38, T2D and MetS)*  *The SMS were well timed and had really good advice. Found it easy to use, and easy to follow up the yes, no, unsure replies. The recommendations I managed to fit as much as I could into my day and try to reinforce anything that I was already doing, and I learned a few other things. I found it very useful (Participant 11, Male, 43, T2D and MetS)*  *Overall, on the frequency I liked it at the start 'cause I think I was just excited for the messages and then I noticed that there were like slowly getting less and I was like It's a rare commodity now (Participant 24, Male, 27, MetS)*  *I like the time, so I was like the first thing I'd see when I'd wake Up. Instead of like Facebook or other messages like other social media messages (Participant 24, Male, 27, MetS)*  *I think the best thing is the time. People can choose the time, so they mean it means that because sometimes I look at my watch at 11:00, it's time for a message (Participant 14, Female, 48, T2D)*  *I found [the phone call] really, really helpful. Just 'cause if I did have any changes, I'd be able to make them, like readily available (Participant 24, Male, 27, MetS)*  Yeah, I think [the phone call] good. ...Like I, I mean, to know that there's a person behind that text message. There's a person behind the trial *(Participant 20, Male, 34, T2D and MetS)* |
| Barriers | - Limited phone capability and data accessibility were identified as barriers to participation for some participants, leading to difficulties in accessing links and responding to messages. For these individuals, these challenges contributed to periods of disengagement. | *My phone, it doesn't do that. I've got a very cheap phone (Participant 1, Male, 55, T2D)*  *Yeah, well, most people I know have an Apple Phone, so I can send text messages just through the data at home. Whereas if it's a phone call or a text message from a non-apple, yeah, I need credit. So yeah, like the links and stuff I can access with my data, it's just responding really that I need the data for (Participant 15, Male, 44, MetS)*  *I just run out of credit one point (Participant 18, Male, 43, MetS)* |
| Suggestions for improvement | A few participants suggested that having all the information compiled into a physical booklet would improve accessibility and usability.   - Increasing interactivity was another recommendation, with some participants highlighting the potential benefits of follow-up text messages or check-in to reinforce key messages and progress and one participant suggested an anonymous online support group for those using the intervention at the same time - The ability to ask questions and receive tailored responses was also highlighted as well as additional texts when a response suggested that the participant might need further support | *I would have liked if all the information had been in one, in a book, it would have been easier to go to, you know, if the text said open up page 63, it would be easy to open that up and read it on paper… then I’d use it more (Participant 2, Female, 49, pre-T2D)*  *Maybe a follow up text or something, maybe like at 8:30 and maybe around lunchtime to remind me… needs another message to help tie in (Participant 5, Male, 43, T2D)*  *Like maybe a second topic of the day, like a second link to a different topic or asking if it wants to continue same topic (Participant 24, Male, 27, MetS)*  *It'd be good if I could ask questions and then, like, they'd respond with, like, more information (Participant 7, Male, 23, MetS)*  *Maybe online support group may cause like you can be anonymous. You don't have to, have a real name or you don't have to have. I don't know how it would work. Say like that, you know? (Participant 17, Male, 44, T2D)*  *If I if I replied a no answer to certain things instead of a yes, then I think that maybe more frequent text messages in a sense that then if you say no then you know that you're not active. Or you're not doing a positive change towards the diabetes or your exercise or your diet management (Participant 17, Male, 44, T2D)* |
| Continuation | - There was a consensus from participants for ongoing support through the intervention. - For those who were less inclined to continue, this was influenced by whether they had a diabetes diagnosis, with those at risk but not diagnosed feeling less need for ongoing engagement. - The preferred duration of continued support varied but was generally tied to having enough time to establish and sustain meaningful lifestyle changes. | *It's not really for me. I'm not diabetic (Participant 3, Male, 42, MetS)*  *Maybe six months, 'cause. I reckon it's a good time for change… I'm thinking if it went longer, you could see the results (Participant 10, Female, 57, T2D)* |
| Helping others | - Participants expressed a strong willingness to recommend SMART to others, highlighting its ease of use, valuable content and effectiveness as a reminder to keep health goals on track. - Some participants noted that the messages were not only informative but engaging which can spark curiosity and further learning. - SMART was also seen to be a preventative measure for those at risk of diabetes. | *It's just good advice, you know… [people] should listen to it. And keep the text messages so that they don't forget what they've been told (Participant 1, Male, 55, T2D)*  *[I would] tell them that it's like super easy to read. Super, super easy to respond. That you can get a lot out of it, a lot of information (Participant 7, Male, 23, MetS)*  *[I would] tell them to try it and just be ready. Because I was like, it's a lot of fun when you first start with the messages and following links, and then you might even write down some information or might even get like giddy and like research, some of this stuff (Participant 24, Male, 27, MetS)*  *Because you know, the medication does weight gain and there's only so much the doctors can do. And the nurses can do, you know, but having a programme like this, it helps because, you know. Not only can you look after your selfcare, but you know you get a few tips here and there about food and what to do. It might be good for people who are also like not social. You know you who aren’t like going to social groups or, you know, they feel alone and they don't know what to do with their their health or they might not have enough family members to encourage them or they might not even have friends to like, help them. So if if they get these booklets and these messages it might, might do good (Participant 29, Female, 29, T2D)*  *If you look at people who have diabetes already, umm, managing it is great. But for people who are prediabetic or people are you know facing. If they're almost at the point where they're diabetic, you might help them, like you might prevent it in in a way, because the more knowledge you have about it, the better you're going to be (Participant 29, Female, 29, T2D)* |
| ***Mechanisms supporting change*** | | |
| *This theme captures how the intervention influenced participants’ actions and well-being and facilitated behaviour change.* | | |
| Learning | - The intervention’s lifestyle-focused content provided many participants with increased awareness, new insights and practical strategies. While some participants were already familiar with certain information, those encountering it for the first time found it particularly relevant and valuable. - Participants appreciated the guidance, fresh perspectives, and practical advice offered. - The content introduced different approaches to making positive changes, giving participants useful ideas for improvement. - Some participants actively saved and revisited the information, finding it a helpful ongoing resource. - Exposure to varying viewpoints encouraged reflection and broadened participants’ understanding of health and lifestyle choices. | *It's nothing I haven't heard before, but if people haven't heard it before, it's good, especially (Participant 3, Male, 42, MetS)*  *I think it was good because it brought a lot of awareness. It made me think consciously about things around to do with my diabetes. It made me feel like, what’s the word? Yeah. Just aware. And with that awareness comes, I’m able to make good decisions which help me with my diabetes management basically (Participant 12, Male, 38, T2D and MetS)*  *Well you are learning something that can assist you so that ah, we all got to be healthy, not to die of a heart attack and not to put any more weight on. Well, that's something that I most desperately need, don't I (Participant 26, Female, 56, T2D and MetS)*  *Sometimes it had information that I didn't know. So, I learnt something (Participant 10, Female, 57, T2D)*  *Think just the information I got. That was the big thing.*  *Because I didn't, I didn't know much about the diabetes because even the doctor doesn't really go into it. He just says that what are your blood sugars like and you tell him then he goes you need to get it down. You need to stop eating chocolate. You need to stop eating sugary stuff. That's hard (Participant 27, Male, 51, T2D and MetS)*  *You know it brought my condition to my attention, and it would point me to places to have a look at, how to create a life of health (Participant 11, Male, 43, T2D and MetS)*  *I learnt [it], so I know how to apply, like, the implication of putting into, like a plan of action, so I can just do that for myself…knowledge is power (Participant 18, Male, 43, MetS)*  *It was interesting, the content and I could go back through them and look up the pages again and yeah, it's just something positive to remind me of something that usually takes granted, like sugar intake….so I've learned a lot from it and it was an easy way to access it, because I can always go to the pages that they recommended and bookmark them (Participant 11, Male, 43, T2D and MetS)*  *I've still got like some of the windows saved on my phone so that I can always go back to them and then if it's like such good helpful tips that like I utilise now (Participant 24, Male, 27, MetS)*  *I think you you did talk about stress management, which is really helpful because like I have my condition is about stress and, you know when you're hungry, when you're stressed out, you eat more. And when you're stressed out, you, you do not control your diabetes as much as you would like. So you know those coping mechanisms that I learned, like how to eat, like try to exercise and eat healthy. It was really good. Yeah (Participant 29, Female, 29, T2D)* |
| Reminders | - Several responses described the intervention as valuable source of reminders that kept their health goals in focus and mitigated distractions of daily life. - For some participants, this was an important prompt for early intervention and encouraged proactive health management. - The intervention and message content reinforced healthy behaviours and supported habit formation. | *I'm not at a point where I'm testing myself every day. But umm but if I don’t look after myself, I know I’ll become diabetic. Umm, it’s part of the pathway I was on and if I didn’t have that reminder I would just you know I might drink more often, or I might you know, take it all in and not realise that impact on my liver. So, you know, I'm just doing damage to myself, and I wouldn't realise that it’s a one-way street, once you’re done, you can only bring it through, you know, remission you can't really get rid of it (Participant 11, Male, 43, T2D and MetS)*  *It's a reminder, every message is a reminder of where I'm at, where I could be, or what I could do so it's an action-based programme (Participant 11, Male, 43, T2D and MetS)*  *I’ve decided to try to get healthier and every day, it's a reminder to stick to my goals, so it helped me it. It was a daily reminder to make better decisions (Participant 9, Male, 46, pre-T2D)*  *It helped me maintain my blood glucose level and reminded me whether, when it was a good time to exercise (Participant 21, Female, 37, T2D and MetS)*  *...remind[s] them to keep on track with their health goals and instead of like getting caught up in the busyness of life… Because you can forget, you can I neglect your health goals when you get busy (Participant 19, Female, 47, MetS)*  *It was, yeah. After, after all these questions I had to answer, I felt pretty. I felt more confident in how to manage my diabetes because of the um.*  *I guess it was like prompts like it prompted me to think about it, and with the with that thought came confidence to manage it basically (Participant 12, Male, 38, T2D and MetS)*  *Completely and like if they didn't prompt me, maybe I wouldn't. I would forget. It's a good reminder to say, look, I need to check my blood sugars (Participant 17, Male, 44, T2D)*  *Just a reminder of if I'm OK. (Participant 21, Female, 37, T2D and MetS)* |
| Readiness for change and motivation | - The intervention supported some participants in their readiness for change, helping them transition from contemplation to action. - Receiving timely prompts and access to relevant resources provided the "push" they needed to move forward, reinforcing motivation and equipping them with practical tools to take meaningful behaviour changes. | *It definitely made me turn me from the thinking about it stage to the actually doing stage show, I think it was beneficial (Participant 9, Male, 46, pre-T2D)*  *[Quitting smoking was] something I was I was wanting to do for a while and then I just needed the push and like more prep. And then when I see the links that it was sending me, I was like, oh, this is really, really helpful information. And I was like, one as an example it takes you to a link and then you click on the link and you just say what you're there for, and I clicked boredom and then it gives you a list of stuff to do when you're bored (Participant 24, Male, 27, MetS)* |
| Positive change | - Overall, participants self-reported meaningful improvements in modifiable lifestyle behaviours.   Most participants described positive change they attributed, at least in part, to the intervention including increased fruit and vegetable intake, healthier portion control and food choices, greater physical activity, weight loss, and smoking cessation, improved sleep, and enhanced mood. | *I've been exercising a lot, I’ve been exercising more so I'm doing an hour on the exercise bike, resistance bands, boxing, so I've increased my exercise by double just during this period and I've improved my diet so I'm trying to incorporate more fruit and veggies (Participant 9, Male, 46, pre-T2D)*  *Well, actually my physical activity has increased in the last three or four weeks. So maybe that's maybe the trial is partly responsible for that (Participant 6, Male, 59, T2D)*  *Consistency with the size and try not to eat too much because yeah, I've been snacking a bit late at night and umm yeah, I've tried to stop eating cookies and stuff and trying to just have cereal or something that's not so sugary (Participant 15, Male, 44, MetS)*  *Definitely positive changes. I’ve lost a lot of weight. I’ve lost 8 kilos (Participant 18, Male, 43, MetS)*  *Yeah, quitting that was probably the… I don't know how I did it. It's like a Hail Mary, but I'm just so glad that I've quit and that I’ve stayed away (Participant 24, Male, 27, MetS)*  *Feel a bit like happier, I think. Because I think it's like, it's like a process that is happening and, how do I say it like, like, it feels like I'm actually getting something done... You know accomplishing (Participant 7, Male, 23, MetS)* |

References

Braun, V., & Clarke, V. (2006). Using thematic analysis in psychology. *Qualitative Research in Psychology, 3*(2), 77–101.

Guest, G., Bunce, A., & Johnson, L. (2006). How many interviews are enough? An experiment with data saturation and variability. *Field Methods, 18*, 59-82.
