# Supplementary material for: Exploring user perspectives on SMART: qualitative study of novel digital intervention targeting metabolic care in schizophrenia and related disorders
Source: BJPsych Open. 2026 Jan 20;12(1):e44. doi: 10.1192/bjo.2025.10954 (PMC12835714; doi:10.1192/bjo.2025.10954)
Supplement: Arnautovska et al. supplementary material 2 — Arnautovska et al. supplementary material [file S205647242510954Xsup002.docx]

**Supplementary Table 2.** Schedule of text messages in the SMART intervention

| Frequency of SMS per week | Month 1  (weeks 0-4) | Month 2  (weeks 4-8) | Month 3  (weeks 8-12) | Total per module  (weeks 0-12) |
| --- | --- | --- | --- | --- |
| CORE MODULES | |  |  |  |
| Module ranked 1^st^ | 2 | 2 | 1 | 20 |
| Module ranked 2^nd^ | 2 | 1 | 1 | 16 |
| Module ranked 3^rd^ | 1 | 1 | 1 | 12 |
| Module ranked 4^th^ | 1 | 1 | 1 | 12 |
| TOTAL per month | 24 | 20 | 16 | 60 |
| OPTIONAL MODULES | |  |  |  |
| Smoking/vaping cessation | 1 | 1 | 1 | 12 |
| Monitoring BGL | 1 | 1 | 1 | 12 |
| TOTAL per month (max) | 8 | 8 | 8 | 24 |

BGL: blood glucose levels
