# Supplementary material for: Exploring user perspectives on SMART: qualitative study of novel digital intervention targeting metabolic care in schizophrenia and related disorders
Source: BJPsych Open. 2026 Jan 20;12(1):e44. doi: 10.1192/bjo.2025.10954 (PMC12835714; doi:10.1192/bjo.2025.10954)
Supplement: Arnautovska et al. supplementary material 3 — Arnautovska et al. supplementary material [file S205647242510954Xsup003.docx]

Supplementary material S1: Interview guide

Schizophrenia and Diabetes Mobile-Assisted Remote Trainer (S.M.A.R.T.): Pilot testing and feasibility study

| **Interview conducted by:** …………………………………………………….. **Date:** ……………  **Participants name:** ………………………………………………………..…  **Phase of the study** (tick one option): ❑ Phase 1 (4-week pilot) ❑ Phase 2 (feasibility study) |
| --- |

**Aim:** Assess the acceptability and feasibility of the S.M.A.R.T. intervention for diabetes self-management among people with schizophrenia.

**Warm-up**: *Thank you very much for agreeing to speak to us about your experience with using the diabetes self-management intervention S.M.A.R.T. that you’ve been receiving over the last 4 [Phase 1] / 12 [Phase 2] weeks through text messages on your mobile phone. We would like to know if you found it* ***helpful*** *in improving some of the diabetes self-care behaviours like your nutrition, physical activity and stress coping. Also, we want to know if you faced any* ***challenging or*** ***difficulties*** *over the last 4 [Phase 1] / 12 [Phase 2] weeks of using S.M.A.R.T.? Before I ask more specific questions, can you please tell us, in your own words, [ask Open question]. Remember, there are no right or wrong answers, and we are only interested in your personal views and experiences.*

**Interview questions**:

1. *OPEN QUESTION: How did you find the whole experience of participating in this study over the past 4 [Phase 1]/12 [Phase 2] weeks*?

***ACCEPTABILITY OF INTERVENTIONS (“personal preference of using the intervention”)***

1. *I now have a few statements that I’d like you to rate on a scale of 1 to 5[show Table 1], where 1 means you completely disagree and 5 means you completely agree.*
   1. *Thank you. Can you tell me more about what did you like about the text messages?*
   2. *Were there any things that you did not like about the text messages?*
   3. *On the whole, would you like to have the option of receiving similar text messages on diabetes self-management in the future?*

***APPROPRIATENESS OF INTERVENTIONS (“an intervention fit”)***

1. *The next a statement is about how appropriate receiving and responding to text messages was for you [show Table 2].*
   1. *[if the answer is 3-Neither agree nor disagree/4-Agree/5-Completely agree] Why do you think receiving text messages to improve self-care behaviours relevant to diabetes would work for you? Are there any reasons that this may, however, not work for you?*
   2. *[if the answer is 1-Completely disagree/2-Disagree/3-Neither agree nor disagree] What are the reasons or aspects of receiving text messages about diabetes self-care behaviours that would not work for you? Are there any aspects of the text messages, however, that may work for you?*

***FEASIBILITY OF INTERVENTIONS (“realistic implementation of the intervention”)***

1. *The last statement asks about how realistic or practical you think receiving and responding to the text messages would be [show Table 3]?*
2. *Thanks. We are nearly at the end. We really appreciate your answers. It seems like you feel that receiving the text messages on a regular basis, over a longer period, would be possible [if answers are 4+]/would not be easy for you [if answers are 1/2]. Can you tell us why is that so?*
3. *FINAL QUESTION: Is there anything else that hasn’t been mentioned so far that you think could be helpful to you in improving self-care behaviours relevant to diabetes risk in this study?*

**Closing up**: Ask the Final question and thank participants for their time and contribution to the project.

**Prompts:**

- *How, in your view, has receiving text messages on your mobile phone helped you in managing your diabetes self-care behaviours?*
- *How, in your view, has receiving text messages made managing your diabetes self-care behaviours more difficult?*
- *How were the last 4 [Phase 1]/12 [Phase 2] weeks different to how you go about self-care behaviours like nutrition and physical activity that help reduce diabetes risk?*
- *Was the experience of participating in this study pleasant or not?*
- *Would you be willing to receive the text messages again, would you recommend them to other people?*
- *Did you have any difficulty accessing the links in the messages?*
- *What are your thoughts on the frequency (number) of the text messages you received during the study?*
- *What are your thoughts on the timing (time of day) of the text messages during the study?*
- *Did you like the check-in phone call? Why yes/no?*
- *How relevant were the topics (nutrition, physical activity, stress coping, weight management, smoking cessation, blood glucose monitoring) to you?*
- *Were there any other topics that were not covered in the text messages that you would have liked to see included?*
- *Did you notice any other changes in your nutrition, weight management, physical activity ot coping with stress?*

Table 1: Acceptability

|  | Completely disagree | Disagree | Neither agree nor disagree | Agree | Completely agree |
| --- | --- | --- | --- | --- | --- |
| **I liked receiving the text messages.** | ➀ | ➁ | ➂ | ➃ | ➄ |

Table 2: Appropriateness

|  | Completely disagree | Disagree | Neither agree nor disagree | Agree | Completely agree |
| --- | --- | --- | --- | --- | --- |
| **Receiving text messages on diabetes self-care behaviours on a regular basis would work for me.** | ➀ | ➁ | ➂ | ➃ | ➄ |

Table 3: Feasibility

|  | Completely disagree | Disagree | Neither agree nor disagree | Agree | Completely agree |
| --- | --- | --- | --- | --- | --- |
| **Responding to text messages about diabetes self-care behaviours on a regular basis seems achievable to me.** | ➀ | ➁ | ➂ | ➃ | ➄ |
