# Supplementary material for: Exploring user perspectives on SMART: qualitative study of novel digital intervention targeting metabolic care in schizophrenia and related disorders
Source: BJPsych Open. 2026 Jan 20;12(1):e44. doi: 10.1192/bjo.2025.10954 (PMC12835714; doi:10.1192/bjo.2025.10954)
Supplement: Arnautovska et al. supplementary material 4 — Arnautovska et al. supplementary material [file S205647242510954Xsup004.docx]

**Supplementation material S2:** Lived-experience perspective on participants’ qualitative accounts of SMART

**Positive Behavioural Changes**

1. Drinks fewer fizzy drinks
2. Eats healthier meals (e.g., more fruit)
3. Implements advice on weighing yourself and measuring portion sizes
4. Learnt how to manage stress, which is linked to better eating habits
5. Weight loss (5 kgs) for one participant
6. Learnt how to manage blood sugar levels

**Benefits of Text Messages**

1. Easy-to-understand messages
   1. Good size of messages
   2. Happy with how clear and simple the messages were (appropriate language)
2. Easy to use (yes/no/unsure)
   1. One participant just started to use a mobile phone and found it feasible
3. Personalised messages
4. Informative messages
   1. Learnt something new
5. Non-judgmental messages (positive tone in responses)
6. Messages/links can be re-read at any point in time
7. Advice is especially useful for those who do not have family members or friends who can help (lack of social support)
   1. *This finding should be a new theme*
8. Good for the prevention of diabetes
   1. Provides guidance and necessary education early on
9. Feels like a real person along with you in your journey

**Recommended Changes to Text Messages/Links**

1. Have fewer messages per week
   1. 4-6 per week were too many; it can feel like homework
   2. Ask how many messages a person is comfortable with
2. Better if information were given in a book rather than text messages
   1. One participant had trouble opening links (data issue)
3. Have pictures for exercises
4. Would like to view stories of others who have gone through similar issues
   1. Act as role models
5. Would like to have these messages over the long term (6 months or longer) to create behavioural changes that are sustained
